# Removal of a gadolinium based contrast agent by a novel sorbent hemoperfusion in a chronic kidney disease (CKD) rodent model

**DOI:** 10.1038/s41598-018-37348-2

**Published:** 2019-01-24

**Authors:** Worapol Ngamcherdtrakul, Jingga Morry, Thanapon Sangvanich, Moataz Reda, Daniel S. Bejan, Glen E. Fryxell, Wassana Yantasee

**Affiliations:** 1 Department of Biomedical Engineering, Oregon Health and Science University (OHSU), Portland, Oregon USA; 2 PDX Pharmaceuticals, LLC, Portland, Oregon USA; 3 Pacific Northwest National Laboratory (PNNL), Richland, Washington USA

## Abstract

Gadolinium based contrast agents (GBCAs) have been linked to toxicity in patients, regardless of having impaired or normal renal function. Currently, no therapy is considered highly effective for removing gadolinium (Gd) from the body. We propose a new strategy to reduce blood Gd content that facilitates whole body removal of Gd using a hemoperfusion system consisting of a cartridge of porous silica beads (Davisil®) functionalized with 1,2-hydroxypyridinone (1,2-HOPO). Herein, we report optimization of the hemoperfusion system using an ex vivo blood and an *in vivo* rat model of chronic kidney disease (CKD). In our ex vivo system, 1,2-HOPO-Davisil outperformed Gambro activated charcoal (AC), which is commonly used in clinical hemoperfusion of aqueous toxins, in terms of Gd capture capacity and rate. In the CKD rat model, the 1,2-HOPO-Davisil hemoperfusion system removed Gd by 3.4 times over the Gambro AC system. 1,2-HOPO-Davisil did not change complete blood counts and common blood biochemistry. Thus, this strategy has great potential for clinical translation to manage GBCAs after magnetic resonance imaging (MRI), before Gd can deposit in the body and cause long-term toxicity. Although gadodiamide was used as a proof of concept model for GBCAs in this study, 1,2-HOPO functionalized mesoporous silica could also capture dissociated Gd and other GBCAs.

## Introduction

Gadolinium based contrast agents (GBCAs) are widely used to improve the detail of magnetic resonance imaging (MRI). In patients with renal insufficiency, GBCAs have been associated with increased risk of developing nephrogenic systemic fibrosis (NSF), a debilitating disease characterized by widespread fibrosis in the skin as well as other organs including the lungs, liver, muscles, and heart^1^. However, recently patients with normal kidney function have also reported side effects that they attributed to toxicity of GBCAs^2,3^. This is thought to be associated with gadolinium (Gd) accumulation in the brain and other tissues^3^ like bone and skin reported in patients^4,5^ and animals^6,7^ with normal renal function. Toxicity of GBCAs has been associated with Gd^3+^ dissociation from the gadolinium chelates. Although linear chelates such as gadodiamide (Omniscan™) and gadopentetate dimeglumine (Magnevist^®^) are less stable than macrocyclic chelates, increasing the risk for dissociation of Gd^3+^ into the blood and tissues^8^, the FDA requires label warnings about the possibility of gadolinium retention in the body for all GBCAs in 2017^9^.

Strategies for Gd removal from the body involving the use of chelators, such as ethylenediaminetetraacetic acid (EDTA), diethylenetriaminepentaacetic acid (DTPA), and deferoxamine have been reviewed^10^. However, none have been highly effective. The authors suggested that DTPA should be more effective than EDTA due to higher binding affinity. In a Nephrogenic Systemic Fibrosis (NSF) patient study, although deferoxamine treatment doubled the urinary excretion of Gd as the treatment dose doubled, the blood levels of Gd remained unchanged^11^.

We propose a new strategy to reduce blood Gd contents, which will facilitate whole body removal of Gd due to equilibrium distribution of Gd from organs to blood using a sorbent hemoperfusion system. Sorbent hemoperfusion system contains a column packed with sorbent material for the removal of toxic chemicals from extracorporeal blood as it flows through the column and the cleaned blood flows back to the body. For the best sorbent material for GBCA removal, we have screened several potential materials based on well-ordered mesoporous silica that was self-assembled with monolayer (SAMMS) of ligands such as acetamide phosphonic acid (AcPhos) and three variations of hydroxypyridinone ligands (1,2-HOPO, 3, 2-HOPO, and 3, 4-HOPO) and found 1,2-HOPO-SAMMS to be the most effective at capturing dissociated and chelated Gd (i.e., gadodiamide and gadopentetate) from blood and dialysate^12^. The 1,2-HOPO as a free ligand has also been shown by others to be a strong and selective f-block chelating agent to treat Gd deposition, outperforming commonly used chelators, such as EDTA and diethylenetriaminepentaacetic acid (DTPA) in mice^13^. Our previous study^12^ was performed in batch contacts. Herein, we developed the 1,2-HOPO into a porous silica bead format suitable for a sorbent hemoperfusion system and optimized the flow conditions using ex vivo blood samples. To evaluate the device *in vivo*, we then developed a rodent model with impaired kidney function and sustained blood levels of Gd similar to those found in patients receiving GBCAs.

## Results and Discussion

### Adsorption capacity of 1,2-HOPO-Davisil for gadodiamide in whole blood

**Figure 1:**
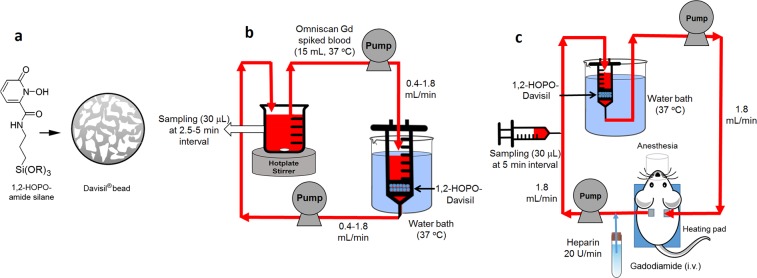
Schematics of 1,2-HOPO grafted on Davisil® beads (**a**) and its use for ex vivo (**b**) and *in vivo* sorbent hemoperfusion systems (**c**). Temperature of extracorporeal blood was controlled to 37 °C with a hotplate stirrer (**b**) or a water bath (**c)**. Total blood volume was 15 mL (4.5 mL in the reservoir where 30 µL of samples were taken as a function of time) in (**b**) and extracorporeal blood volume was 5.0 mL in (**c**). Davisil bead drawing (**a**) was created by W.Y., and (**b**) and (**c**) were created by M.R. using free stock images.

**Figure 2:**
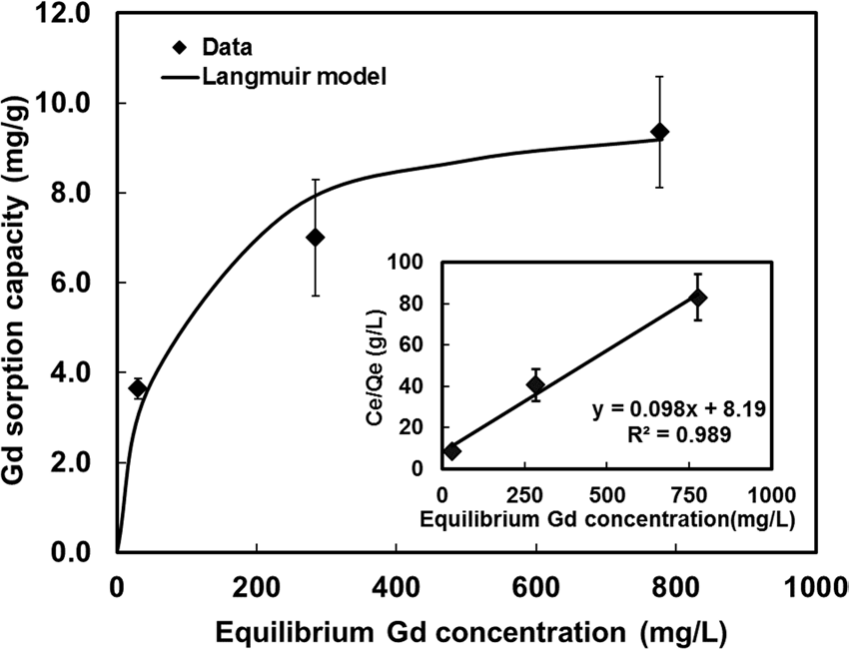
Adsorption isotherm of gadodiamide on 1,2-HOPO-Davisil, measured in heparinized rat blood. Inset shows linear isotherm parameters. Initial concentrations of gadodiamide in blood of 70, 360, or 870 mg/L as Gd and sorbent per blood volume of 0.01 g/mL. Data are reported as mean ± SD (n = 3).

Figure 1a shows the final product (1,2-HOPO-Davisil) developed for sorbent hemoperfusion. Figure 2 shows the adsorption isotherm of gadodiamide on 1,2-HOPO-Davisil measured in heparinized rat blood. The data fit a Langmuir isotherm model well (R^2^ of 0.989) and the maximum adsorption capacity of gadodiamide on 1,2-HOPO-Davisil was predicted to be 10.2 mg Gd (or 38 mg of gadodiamide) per gram of 1,2-HOPO-Davisil. We also confirmed that the 1,2-HOPO-Davisil removed gadodiamide from blood of rats and humans in the same manner and regardless of anticoagulants used (0.38 wt.% Na-acetate or 50 U/mL Na-heparin).

### Ex vivo gadolinium removal by sorbent hemoperfusion systems

**Figure 3:**
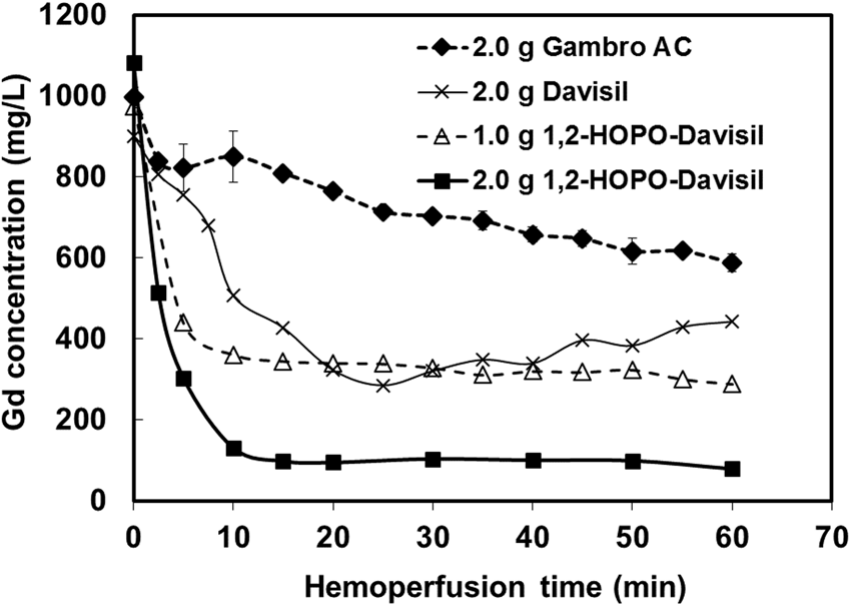
Gadodiamide removal from 15 mL of whole blood using hemoperfusion systems containing various sorbents (mass as specified). Flow rate at 1.3 mL/min.

Gadodiamide removal was performed with hemoperfusion systems containing various sorbents in a closed-loop flow system (15 mL blood total) as shown in Fig. 1b. Figure 3 shows the levels of gadodiamide (measured as Gd) in the reservoir (containing 4.5 mL of blood at the steady state) as a function of time with the zero time point beginning when the first drop of blood had returned from the sorbent bed to the reservoir. Gadodiamide Gd concentration in blood reduced as a function of time that the blood was flowed through the sorbent column. Because error was low for the ex vivo systems (e.g., see the Gambro AC data from n = 2/group), to minimize rat blood use, we performed a single test for the other materials. Among 1,2-HOPO-Davisil, unmodified Davisil, and Gambro charcoal, all at 2.0 g each, 1,2-HOPO-Davisil performed the best. It reduced the blood gadodiamide in the reservoir by about 90% in 10 min. The removal extent remained at 90% from 10 to 60 min, indicating no leachate of gadodiamide from the 1,2-HOPO groups once bound as well as no leachate of the 1,2-HOPO from the Davisil beads. Without 1,2-HOPO functionalization, Davisil beads alone removed gadodiamide by 68% at most by 25 min, but slowly lost the gadodiamide back to the blood (e.g., 50% removal at 60 min), suggesting some physisorption of gadodiamide with the silica substrate. Activated carbon materials have been shown to successfully capture GBCAs from aqueous solutions and urine^14^. In blood, the commercial Gambro activated charcoal (AC) was worst among the three test groups at removing gadodiamide in terms of removal rate and extent. It removed Gd slowly and reached only 41% removal after 60 min. The removal of gadodiamide by 1,2-HOPO-Davisil increased with increasing sorbent amounts from 1.0 g to 2.0 g. One gram of 1,2-HOPO-Davisil also performed better than two grams of unmodified Davisil and Gambro AC.

Figure 3 shows comparative performance among materials. One limitation is that the volume ratio of blood in the reservoir per the total volume was low. Gd reduction rate in the reservoir would be slower than those in Fig. 3 if there was more blood in the reservoir, but faster with the increasing flow rate (faster mixing). Nevertheless, the system would reach the same equilibrium Gd removal by one hour. Evidently, the maximum Gd removal was 10.3 mg/g at the end of hemoperfusion, which was in perfect agreement with the maximum capacity of the material (10.2 mg/g) predicted by the model in Fig. 2.

**Figure 4:**
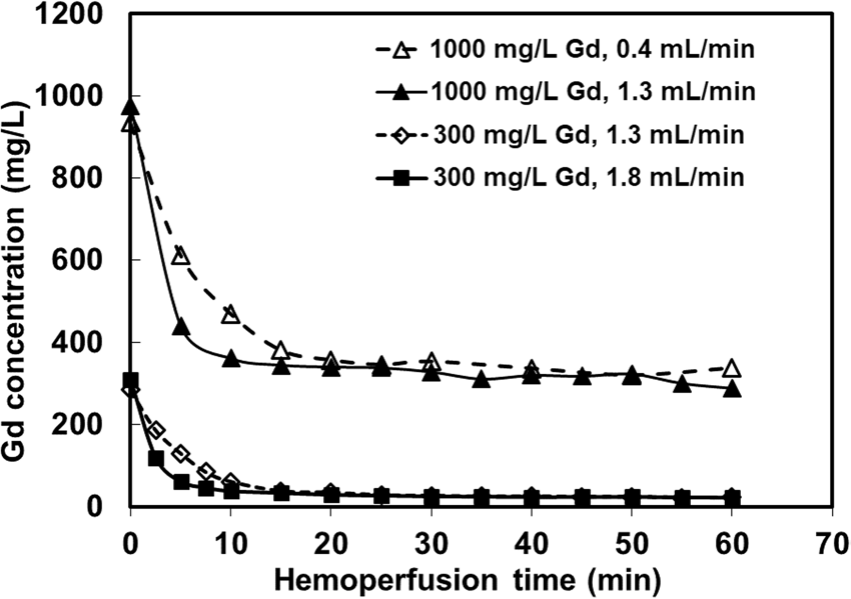
Gadodiamide removal from 15 mL whole blood using a hemoperfusion system containing 1.0 g of 1,2-HOPO-Davisil at specified initial gadodiamide concentrations and flow rates.

Blood flow rate had an effect on gadodiamide removal by sorbent hemoperfusion as shown in Fig. 4. The higher the flow rate, the faster the removal was until the system reached saturation adsorption capacity. This agreed with fast sorption kinetics we previously reported on 1,2-HOPO modified on another silica substrate (MCM-41)^12^. Even at low gadolinium concentration (300 mg/L) having lower concentration gradient than at high gadolinium concentration (1000 mg/L), the faster flow rate (1.8 mL/min) still performed better than the slower flow rate (1.3 mL/min). A flow rate faster than 1.8 mL/min was not feasible with our current setup since it led to bubbles in the sorbent beds. Hence the flow rate of 1.8 mL/min was adopted for subsequent *in vivo* hemoperfusion studies. Although our ex vivo flow experiments were performed in an air-exposed system, we did not observe interference from bicarbonate as a result of CO_2_ exposure; i.e., same maximum Gd capture by 1,2-HOPO-Davisil from this system versus a closed system in Fig. 2. This is not surprising since the effect would be low with gadodiamide Gd, which is less likely to complex with bicarbonate than the dissociated Gd^3+^ counterpart. Even with Gd^3+^ in dialysate containing 32 mEq/L of HCO_3_^−^ (pH 7.6–8.0), we previously showed that 1,2-HOPO on mesoporous silica was more effective for removing Gd^3+^ (K_d_ ~ 10^6^ to 10^7^) than activated carbon (K_d_ ~ 10^3^) and zirconium phosphate (K_d_ ~ 10^2^)^12^.

### Chronic kidney disease (CKD) model

Since gadodiamide toxicity has been more commonly found in CKD patients, we developed a CKD rat model by oral gavage of adenine to male Wistar rats for 12 doses. We achieved significantly elevated serum levels of blood urea nitrogen (BUN) and creatinine in these rats as shown in Table 1, indicating impaired kidney function. The glomerular filtration rate (GFR) of these rats was 17% compared to that of normal kidney rats (GFR_0_), which is equivalent to human stage 4 CKD (having GFR/GFR_0_ of 0.14–0.26^15^).

**Table 1:** Characteristics of adenine-induced CKD rat model created by 12 days dosing of 600 mg/kg adenine to 9 weeks old male Wistar rats. Data are reported as mean ± SD (n = 6).

Group	Body weight (g)	Serum BUN^†^ (mg/dL)	Serum creatinine (mg/dL)	Serum P (mg/dL)	Serum Ca (mg/dL)
Normal rats (pre-adenine dosing)	270 ± 8	9.6 ± 1.9	0.2 ± 0.0	10.9 ± 0.7	11.1 ± 0.5
CKD‡ rats	268 ± 22	170 ± 15	3.3 ± 0.2	11.4 ± 1.7	10.3 ± 2.0

^†^Blood urea nitrogen.

^‡^Chronic kidney disease.

### CKD rat model with prolonged gadodiamide circulation

**Figure 5:**
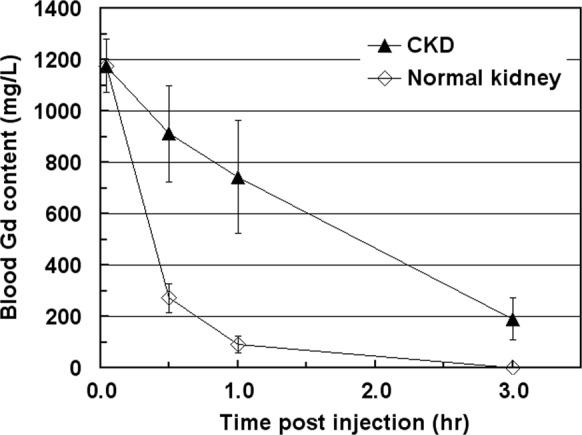
Blood Gd contents of CKD and normal kidney rats measured at various time points post injection with 2.5 mmol gadodiamide/kg. Data are reported as mean ± SD (n = 6 for CKD and n = 4 for normal kidney rats).

Next, we injected the CKD rats with 2.5 mmol gadodiamide/kg via tail vein and monitored the blood Gd concentrations at various time points post injection using ICP-MS as shown in Fig. 5. The impaired kidney function of the CKD rats resulted in prolonged circulation time of Gd compared to that in normal kidney rats. Specifically, CKD rats retained about 3–7-fold higher blood Gd contents at 0.5 and 1.0 hr post injection and 150-fold at 3.0 hr post injection than those of normal rats.

### Removal of gadodiamide ***in vivo*** using sorbent hemoperfusion

**Figure 6:**
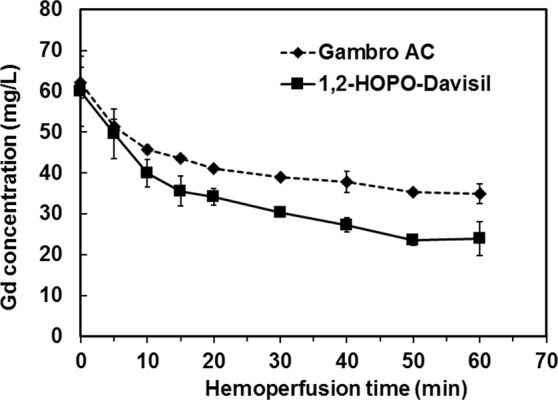
Removal of gadodiamide (as Gd) from CKD rats injected with 0.1 mmol gadodiamide/kg using hemoperfusion systems containing 1.0 g of 1,2-HOPO-Davisil or Gambro AC, at a blood flow rate of 1.8 mL/min for 60 min. Data are reported as mean ± SD (n = 2).

We used the CKD rats with prolonged blood Gd circulation time for our sorbent hemoperfusion studies. In patients with severely reduced renal function receiving 0.1 mmol gadodiamide/kg, blood Gd decreased from 550 to 250 µmol/L (40–90 mg Gd/L) at the time lapse of 1 hr to 24 hr post injection^16^. To achieve similar blood Gd levels, we injected the CKD rats with 0.1 and 0.5 mmol gadodiamide/kg which resulted in initial blood Gd concentrations of about 60 and 330 mg Gd/L, respectively (Table 2). The rats were then subjected to sorbent hemoperfusion as shown in Fig. 1c, and the Gd removal (based on Gd captured on the sorbents) per gram of the sorbent was reported in Table 2. Of the 0.1 and 0.5 mmol gadodiamide/kg injected doses, 33% and 21% was removed with 1,2-HOPO-Davisil, respectively. The removal was about 3.4 times greater than that by the Gambro AC counterpart. In the end, 1,2-HOPO-Davisil reduced blood Gd content of the rats injected with 0.1 mmol gadodiamide/kg to 24 mg/L, while Gambro AC reduced to 35 mg/L as shown in Fig. 6.

**Table 2:** Removal of gadodiamide (as Gd) from CKD rats injected with 0.1–0.5 mmol/kg gadodiamide using hemoperfusion systems containing 1.0 g of 1,2-HOPO-Davisil or Gambro AC, blood flow rate of 1.8 mL/min for 60 min. Data are reported as mean ± SD (n = 2).

Gadodiamide dose	Sorbent	Initial blood Gd concentration (mg/L)	Gd removal	
			mg Gd/g sorbent	% Gd removal
0.1 mmol/kg	Gambro AC	62 ± 3.7	0.35 ± 0.05	9.4 ± 1.3
	1,2-HOPO-Davisil	63 ± 9.7	1.45 ± 0.3	33 ± 6.5
0.5 mmol/kg	Gambro AC	318 ± 16.7	1.31 ± 0.25	6.50 ± 1.13
	1,2-HOPO-Davisil	341 ± 35.5	4.31 ± 1.47	21.3 ± 5.77

### Safety of HOPO-sorbent hemoperfusion

Blood biocompatibility tests ex vivo showed no significant changes in complete blood count parameters with 1,2-HOPO-Davisil compared to the system without sorbent (i.e., control blood) or Gambro AC, as shown in Table 3. One exception is the plasma glucose, which was significantly reduced in the Gambro AC group compared to that of the control blood and 1,2-HOPO-Davisil. Hypoglycemia (low blood glucose) is one complication reported for hemoperfusion with activated charcoal^17^, which can capture glucose nonspecifically due to its high surface area. In rats, hemoperfusion with 1,2-HOPO-Davisil was well tolerated at 1.8 mL/min of blood flow rate. No blood clotting was observed during the one hour of hemoperfusion, and the rats recovered well after hemoperfusion. Table 4 shows that removal of essential minerals such as Na, P, K, Ca, Fe, and Zn by 1,2-HOPO-Davisil was not significantly different than by Gambro AC (p-values > 0.05) with the exception of Na, Mg, and Cu (p-values < 0.05). Nevertheless, both materials removed less than 1.0 mg of these metals per gram of sorbent with the exception of Na (about 1.2 mg/g). The serum levels of inorganic P, Ca, BUN, and creatinine were also not significantly different than those before hemoperfusion by 1,2-HOPO-Davisil as shown in Table 5. Overall, our preliminary safety studies indicate that hemoperfusion with 1,2-HOPO-Davisil has as good safety profile as (if not better than) charcoal AC.

**Table 3:** Hematology tests to assess the effects of 1,2-HOPO-Davisil and Gambro AC on blood characteristics.

	Control Blood	1,2-HOPO-Davisil	Gambro AC (Adsorba®)
Leukocyte Count (10^3^/µL)*	5.41 ± 0.22	5.49 ± 0.02	5.66 ± 0.26
Erythrocyte Count (10^6^/µL)	5.22 ± 0.07	5.61 ± 0.33	5.22 ± 0.27
Hemoglobin (g/dL)	15.0 ± 0.3	15.8 ± 1.0	15.0 ± 0.6
Hematocrit (%)	44.5 ± 0.1	48.0 ± 3.0	44.3 ± 2.0
Mean Corpuscle Volume (fL)	85.3 ± 1.4	85.7 ± 0.5	84.9 ± 0.6
Mean Corpuscular Hemoglobin Concentration (g/dL)	33.7 ± 0.7	32.9 ± 0.0	33.8 ± 0.1
Platelet count (10^3^/µL)*	202 ± 9.9	217 ± 2.5	194 ± 4.0
Mean Platelet Volume (fL)	10.0 ± 0.6	10.3 ± 0.8	10.0 ± 0.1
Plasma glucose (mg/dL)	65 ± 5	65 ± 2	35 ± 0^#^

Heparinized blood (15 mL, 37 °C) was flowed at the rate of 1.4 mL/min for 60 min. Data are reported as mean ± SD (n = 2).

*Performed in batch experiments with whole blood containing EDTA as the anticoagulant.

^#^Significantly different (p < 0.05) from control blood and 1.2-HOPO-Davisil.

**Table 4:** Removal of other metals from blood of CKD rats from the same experiments as described in Table 1.

Sorbent	Amount of other metals (mg) captured on 1.0 g of the sorbent							
	Na#	Mg#	P	K	Ca	Fe	Cu#	Zn
Gambro AC	0.37 ± 0.24	0.09 ± 0.02	0.01 ± 0.01	0.00 ± 0.00	0.32 ± 0.06	0.06 ± 0.07	0.01 ± 0.00	0.01 ± 0.01
1,2-HOPO-Davisil	1.17 ± 0.29	0.67 ± 0.14	0.04 ± 0.08	0.03 ± 0.07	0.66 ± 0.29	0.05 ± 0.06	0.00 ± 0.00	0.01 ± 0.00

Data are reported as mean ± SD (n = 4).

^#^Significantly different (p < 0.05) between Gambro AC and 1,2-HOPO-Davisil.

**Table 5:** Effects of hemoperfusion with 1,2-HOPO-Davisil on serum biochemistry of rats.

Group	Serum BUN^†^ (mg/dL)	Serum creatinine (mg/dL)	Serum P (mg/dL)	Serum Ca (mg/dL)
Before hemoperfusion	170 ± 15	3.3 ± 0.2	11.4 ± 1.7	10.3 ± 2.0
After hemoperfusion	173 ± 15	3.4 ± 0.4	12.3 ± 3.1	11.4 ± 1.3

Data are reported as mean ± SD (n = 6).

^†^Blood urea nitrogen.

## Conclusion

We successfully developed a rat model with impaired kidney function, which retained gadodiamide in the blood longer than the normal kidney rats, providing a window for preclinical evaluation of our 1,2-HOPO-Davisil hemoperfusion system. Our system is 3.4 times more effective than the commercially available Gambro activated charcoal used for toxic metal removal from patients’ blood. It is also shown to be as safe as the Gambro AC, thus has great potential for clinical translation. Removal of gadodiamide Gd from blood would result in reduced Gd retention in tissues and organs and may decrease the risk of NSF occurrence in end-stage CKD patients as well as gadolinium related toxicities recently reported in some normal kidney patients receiving multiple MRIs with GBCAs. Although the work employed gadodiamide, 1,2-HOPO-Davisil should also be effective at capturing dissociated Gd (Gd^3+^) and gadopentetate (Magnivist) as previously reported by us on batch sorption studies of 1,2-HOPO on MCM-41^12^.

The adenine induced CKD rats with a single gadodiamide dose have the advantage of better representing the clinical conditions and characteristics of NSF patients compared to the normal kidney rats. Despite the observed Gd deposition in various tissues, there remains unclear knowledge on the mechanisms of Gd-induced toxicity, which need to be elucidated in order for therapeutic intervention strategies to be successfully developed. Potential mechanisms of Gd toxicity include the induction of oxidative stress, apoptosis, transmetallation^2^ and competition with endogenous Ca^2+^ ions in biochemical processes, due to its similar size with Gd^3+^, resulting in the disruption of many calcium-dependent enzymes^18^. The adenine-induced CKD rat model has greater long-term survival rate than the standard 5/6-nephrectomy model, thus long-term monitoring of Gd deposition and its effects is possible with this model.

## Materials and Methods

### 1,2-HOPO functionalized Davisil beads

Benzyl protected 1,2-HOPO ligand was synthesized following the published procedure^19^. High-purity grade Davisil® 636 (pore size 60 Å, surface area of 500 m^2^/g, 150–250 micron bead size, Sigma-Aldrich) was used as the substrate. For ligand deposition on the Davisil beads, we first synthesized the benzyl protected 1,2-HOPO-amide silane following our previous published protocol^20^. Next, Davisil beads (42 g) were then suspended in toluene and treated with sufficient water (6.28 mL) to give 10^19^ water molecules per square meter (roughly 2 monolayers worth). This slurry was stirred at room temperature for one hour. Then 34 mmol 1,2-HOPO amide-silane solution was added to the toluene-Davisil slurry with vigorous stirring, and heated to reflux for 4–6 hours. The material was then filtered, washed with 2-propanol, and air-dried. Deprotection was accomplished by treating the material with 10% HBr in acetic acid and stirring the suspension overnight at ambient temperature. The product (1,2-HOPO-Davisil, see Fig. 1a) was filtered out, washed with 2-propanol, and air-dried. Approximately 0.5–1.0 silanes/nm^2^ was achieved with this method by solid-state ^29^Si NMR.

### Gadolinium removal from ex vivo blood

Gadolinium capture from blood was performed in batch and in flow experiments. For batch experiments (adsorption isotherm), rat whole blood containing 0.38 wt.% sodium citrate or 50 U/mL heparin was spiked with gadodiamide to achieved various concentrations (70–900 mg/L as Gd) and incubated for 30 min before use. 1,2-HOPO-Davisil was added to the Gd spiked blood at 0.01 g/mL and the mixture was shaken at 200 rpm at 37 °C for 2.0 hr. Afterward, the beads were filtered out using a filter made in-house consisting of a stainless steel screen (mesh size of 100 µm), and the filtered blood was digested and subjected to metal analysis with inductively coupled plasma-mass spectrometer (ICP-MS).

For flow experiments (ex vivo hemoperfusion, schematic as shown in Fig. 1b), 1.0 or 2.0 g of 1,2-HOPO-Davisil beads (150–250 µm, Fig. 1a) were packed in a plastic housing between two stainless steel screens (mesh size of 100 µm). Gadodiamide-spiked rat blood was flowed from a blood reservoir (continuously stirred at 350 rpm and heated to 37 °C, using a thermometer to monitor temperature of blood in the reservoir) through the column bed at the flow rate of 0.4–1.8 mL/min and back to the reservoir in a closed loop manner using two peristatic pumps. The total blood volume in the system was 15 mL, at the steady state, consisting of 4.5 mL in the reservoir, 8 mL in the tubing upstream from the sorbent bed and in the column combined, and 2.5 mL in the tubing downstream from the sorbent bed. Blood specimens (30 µL each) were sampled with a pipette (see Fig. 1b) directly from the reservoir at various time points from 0 to 60 minutes for ICP-MS analysis of metal contents. The zero time point started when the first drop of blood was back from the sorbent bed to the reservoir. Experiments were repeated with the same mass of Davisil beads (without 1,2-HOPO) or activated charcoal (Adsorba®, Gambro, Sweden), commonly used in human hemoperfusion systems.

### Inductively coupled plasma-mass spectrometer (ICP-MS) analysis

Blood was digested in concentrated nitric acid with heating at 65 °C until fully digested. Samples were then diluted 25-fold with DI water, and subject to metal analysis with ICP-MS (Agilent 7700X, Agilent Technologies, Santa Clara, CA).

### Adenine-induced chronic kidney disease (CKD) rat model

Male Wistar rats (9 weeks old, n = 6/group) were orally gavaged (between 8:00 and 9:00 am) with adenine at a dose of 600 mg adenine/kg body weight/day for 12 consecutive days except weekends. The adenine suspension was prepared from adenine hemisulfate salt (Aldrich Co) in saline (0.9% NaCl) solution at a concentration of 100 mg/mL. The controls received oral gavage of saline solution containing no adenine and were food matched with the adenine dosed group. Rats were weighed daily and food intake was recorded. Blood samples were collected from the saphenous vein between 12:00–1:00 pm on days 5 and 10 of gavage as well as weekly thereafter. All animals were maintained on a 12 hr (6 am to 6 pm) light schedule and given water ad libitum. All animal experiments were approved by the OHSU’s Institutional Animal Care and Use Committee (IACUC) and were carried out under the auspices of the OHSU Department of Comparative Medicine.

### Blood compatibility

To evaluate the biocompatibility of sorbent materials, we used fresh human whole blood collected from healthy adult donors with informed consents and approval by OHSU’s Institutional Review Board for Protection of Human Subjects (IRB). The blood (15 mL) was anticoagulated with 50 U/mL heparin, and subjected to a flow experiment similar to Fig. 1b, except without the use of the reservoir. Blood flowed (1.4 ml/min, and heated to 37 °C, using a thermometer to monitor temperature) through a column bed packed with either 1.0 gram of 1,2-HOPO-Davisil or 1.0 gram of Gambro AC, in a plastic housing between two stainless steel screens (mesh size of 100 µm). After 1 hour, 3 mL and 2 mL of blood was collected for hematology (complete blood count, Sysmex XN-10 analyzer) and glucose testing (Siemens Dimension Vista 1500), respectively. Because platelets and leukocytes can clump in heparin, causing erroneous counts^21^, we conducted another experiment using whole blood containing EDTA as the anticoagulant. Specifically, fresh human whole blood from healthy volunteers was collected in K3 EDTA blood collection tubes (BD vacutainer, Franklin Lakes, NJ) and was subjected to batch experiments, using 0.5 g of 1,2-HOPO-Davisil or Gambro AC in 3.0 mL of blood, shaken at 70 rpm at 37 °C, for 1 hour. A control group with no sorbent material or Gambro AC was also performed in parallel.

### Serum biochemistry

Serum levels of blood urea nitrogen (BUN), creatinine, calcium and inorganic phosphorus were measured with the DRI-CHEM 4000 Chemistry Analyzer (HESKA, Loveland, CO). The detection ranges were 5–140 mg/dL for BUN, 0.2–24 mg/dL for creatinine, 0.5–15 mg/dL for inorganic phosphorus, and 4.0–16.0 mg/dL for calcium.

### Kidney function assessment

Glomerular filtration rates (GFR) of normal kidney rats and adenine-induced CKD rats were estimated based on creatinine clearance. The 24-hr urine of each rat was collected and analyzed for creatinine, while a spot blood sample was collected from the same time period and analyzed for serum creatinine. The urine creatinine was measured using Quantichrome™ Creatinine Assay Kit (Hayward, CA) with the detection range of 0.1–50 mg/dL; while the serum creatinine was analyzed with the DRI-CHEM 4000 Chemistry Analyzer as previously described.

### Gadodiamide pharmacokinetics

Gadodiamide was injected tail-vein to male Wistar rats (9 weeks old, n = 4–6/group) once at a dose of 2.5 mmol/kg body weight, and blood samples were taken from saphenous vein at various time points from 2.5 min to 3 hr post injection. The Gd contents of blood samples were determined by ICP-MS as previously described.

### ***In vivo*** sorbent hemoperfusion

The adenine-induced CKD rats (2 per group) with double venous cannulation were injected with a single dose of 0.1–0.5 mmol gadodiamide/kg body weight and immediately connected to a sorbent hemoperfusion device to remove the gadodiamide. Schematic of the experimental setup is shown in Fig. 1c. One gram of 1,2-HOPO-Davisil or Gambro Activated charcoal (AC) was used in the hemoperfusion system, constructed the same way as the ex vivo hemoperfusion experiments. Temperature of the whole system was controlled at 37 °C using a water bath. Blood was pumped out from the rats through the sorbent hemoperfusion and back to the rats at the flow rate of 1.8 mL/min throughout. An anticoagulant Heparin was pumped to the blood extracorporeal loop at the rate of 20 U/min. Total hemoperfusion time was 60 min. Blood specimens (30 µL) were collected at 5-minute intervals from the cannula and subjected to ICP-MS analysis as previously described. The rats were under anesthesia and the body temperature was monitored throughout the hemoperfusion process. After completion of sorbent hemoperfusion (60 min), the sorbent was flushed with DI water to remove the unbound Gd. Then the bound Gd was leached out from the sorbent using 3 batches (20 mL, 10 mL, and 10 mL) of 10 M HNO_3_ until no further Gd leached out. The Gd contents of each acid batch were analyzed by the ICP-MS and the total Gd leached out was reported per gram of sorbent.

### Statistical analysis

Differences between the mean values of two groups were analyzed by one way ANOVA or Student’s t-test. Results were considered statistically significant at p-value < 0.05.

## Data Availability

The data supporting the findings of this study are available within the paper and are available from the corresponding author upon request.
